# Remarkable Increase in the Prevalence of Overweight and Obesity Among School Age Children in Antalya, Turkey, Between 2003 and 2015

**DOI:** 10.4274/jcrpe.galenos.2018.2018.0108

**Published:** 2019-02-20

**Authors:** Gamze Çelmeli, Yusuf Çürek, Zümrüt Arslan Gülten, Mehmet Yardımsever, Mustafa Koyun, Sema Akçurin, İffet Bircan

**Affiliations:** 1 University of Health Sciences Antalya Training and Research Hospital, Clinic of Pediatric Endocrinology, Antalya, Turkey; 2 Akdeniz University Faculty of Medicine, Department of Pediatrics, Antalya, Turkey; 3 Akdeniz University Faculty of Medicine, Department of Biostatistics and Medical Informatics, Antalya, Turkey; 4 Akdeniz University Faculty of Medicine, Department of Pediatric Endocrinology, Antalya, Turkey

**Keywords:** Obesity, prevalence, school age children, Turkey

## Abstract

**Objective:**

Childhood obesity (OB) is an acknowledged global problem with increasing prevalence reported around the world. We conducted this study with the aim of determining the local trend in OB and overweight (OW) prevalence in the last decade and to observe the alteration of OB and OW prevalence by age group. An additional aim was to construct new age- and gender-specific body mass index (BMI) reference percentile charts for Turkish children living in the city center of Antalya.

**Methods:**

This cross-sectional study included 1687 school aged children. International Obesity Task Force guidelines were used to determine the OB and OW prevalence. OW was defined as a BMI between 85^th^ and 95^th^ percentile, and OB >95^th^ percentile. The data were compared with a previous study carried out in the same region in 2003. The least mean square method was used to construct the BMI reference percentile charts.

**Results:**

The prevalence rates for OB and OW were 9.8% and 23.2%, respectively, with a combined OW/OB rate of 33%. OB prevalence was higher in boys than girls (p<0.05). The prevalence of combined OW/OB was highest at age 9-10 years. The prevalence of OB has increased 2.9 times during twelve years in this location.

**Conclusion:**

Comparing the current findings with rates of OW and OB in the previous decade, childhood OB in Antalya has reached alarming levels. Urgent measures integrated into the national education system should be taken to prevent OB. In addition more surveillance studies should be planned to show the future trend of OB prevalence nationally.

**What is already known on this topic?**

The increase in prevalence of obesity and overweight among children and adolescents is a major global public health problem in both developed and developing countries.

**What this study adds?**

This is a 12-year interval study referring to our previous study conducted in 2003. Our findings suggest that the prevalence of OW and OB in school-age children living in the same geographical region of Turkey has increased around three-fold.

## Introduction

During the last few decades, the number of obese (OB) and overweight (OW) children and adolescents has significantly increased in both developed and developing countries. This change poses a major public health threat, globally (1). 

From 1980 to 2013, the prevalence of combined OW/OB among children and adolescents in developed countries has risen from 16.9% to 23.8% in boys and from 16.2% to 22.6% in girls. In developing countries, the prevalence at these ages has also increased from 8.1% in 1980 to 12.9% in 2013 among boys, and 8.4% to 13.4% among girls (2). In 2016, it was estimated that 50 million girls and 74 million boys worldwide were OB (3).

Although there are many reports from different regions of Turkey, there is no nationwide systematic study investigating OB trends in Turkish children. Alper et al (4) reported, in a meta-analysis of 58 publications from Turkey, an increase in prevalence of OB from 0.7% in 1990-1995 to 7.1% in 2011-2015 (1.2% to 6.8% for girls, 0.3% to 7.4% for boys). Bereket and Atay (5) reported that OW and OB prevalence was higher in the western regions of Turkey where the population generally has a higher socioeconomic status (6).

The primary aim of this study was to determine the prevalence of OW and OB among school aged children in Antalya, Turkey, and to compare our data with those of a similar study conducted in 2003 in the same region (7). The data from this study will also enable the creation of age- and sex-specific body mass index (BMI) reference percentile charts and BMI curves for Turkish children living in the city center of Antalya.

## Methods

Data collection for this cross-sectional study was carried out in March-April 2015. The study included children from 58 out of the 124 schools located in the Muratpaşa district of Antalya city, a district with a relatively high socio-economic level population. From a total of 61092 school children, 1687 healthy children (873 boys and 814 girls) aged between 6-14 were selected for the study using a population-based, stratified, cluster-sampling method. 

Written permits for the study were obtained from Antalya Provincial Directorate of Health and Antalya Province National Education Directorate. Informed consent was obtained from all students and their parents. The study was approved by the Ethics Committee of Akdeniz University (decision no: 108, date: 25.02.2015).

The ages of all participants were calculated from the day of data collection according to their date of birth to calculate decimal age. Decimal ages were grouped in years, for example; 6 years (6-6.99 years). Weight was measured with light clothes and without shoes, using a digital portable scale and was rounded up to the nearest 100 g. Height was measured with the subjects standing in the Frankfurt plane, using a laser rangefinder (BOSCH, Leinfelden-Echterdingen, Germany) calibrated with Harpenden stadiometer, sensitive to the nearest 0.1 cm. BMI was calculated as weight/ height² (kg/m²). Age and gender specific International Obesity Task Force references as defined by Cole et al (8) were used to determine the prevalence of OW and OB. OW was defined as BMI between 85th and 95th percentile and OB as BMI above the 95th percentile. To be able to perform comparisons with the current study, the data of 1775 children aged 6-14 years were selected from a previous study with adjustments for age and sex (7).

### Statistical Analysis

Data were analyzed using the Statistical Package for Social Sciences version 22 software (IBM Inc., Chicago, Ill., USA). Differences between categorical variables were tested by the Pearson and Fisher chi-square tests, while BMI values were compared with the z-test.

We used the LMS method to construct age and gender specific BMI reference percentile charts and BMI curves for Turkish children living in the city center of Antalya (9). In this method, L represents the skewness, M represents the median and S represents the coefficient of variation of the data. The BMI centile curves were smoothed by using the distance-weighted least squares procedure.

## Results

**Figure 1:**
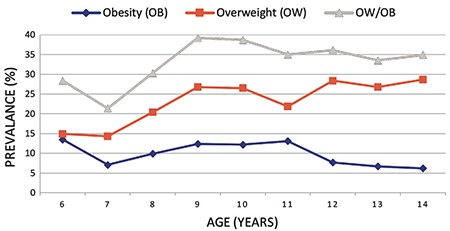
The prevalence of obesity and overweight combining both genders in by age group, in Antalya, Turkey

**Figure 2:**
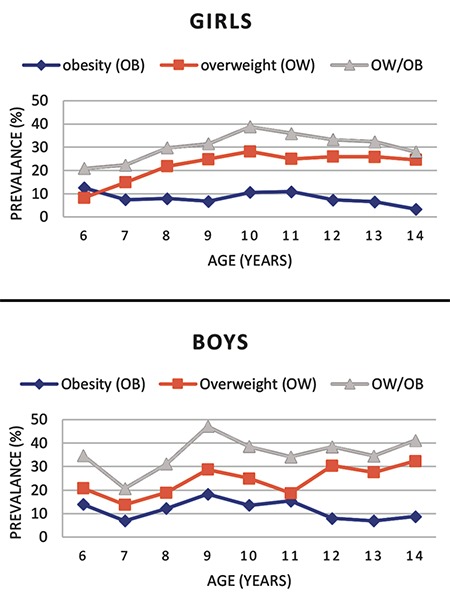
The prevalence of obesity and overweight among girls and boys by age group in Antalya, Turkey

The overall prevalence of combined OW/OB in the current study was 33 %, while the prevalence figures for OB and OW were 9.8% and 23.2%, respectively. There was no significant difference between boys and girls for OW prevalence. However, OB prevalence was higher in boys (11.3%) than in girls (8.1%) (p<0.05; see Table 1). The prevalence of combined OW/OB was also higher in boys (35.2 %) than in girls (30.6 %). The distribution of OB and OW prevalence for all children according to age group is shown on Figure 1. We observed that the prevalence of combined OW/OB increased rapidly from seven years to nine years of age (p<0.05), formed a plateau between the ages of nine and 10 years and then decreased from the age of 10 onwards. The prevalence of OB and OW by age group is depicted in Figure 2 for each gender separately. The prevalence of combined OW/OB was found to increase with age between six and 10 years among girls (p<0.05), while a rapid increase in prevalence was found from seven to nine years in boys (p<0.05). The peak prevalence of combined OW/OB was at 10 years of age in girls (38.8%) and at nine years in boys (47.1%). The prevalence of OB alone was not statistically significant when age groups were compared.

Mean BMI (± standard deviation) values and the cutoff points of BMI, obtained by using the LMS method for OW and OB (85^th^ and 95^th^ percentiles) by age group are shown in Table 2. The BMI centile curves were also generated using the LMS method (data not shown).

The prevalence of combined OW/OB was found to increase up to 1.8-fold (from 18% to 33%) from 2003 to 2015, while OB prevalence showed a 2.9-fold increase during the same period (3.4% to 9.8%) (Table 3).

## Discussion

This study has merit because it is one of the most recent studies in Turkey investigating OW and OB prevalence and its trend among school children residing in the same geographical region by age and sex. This study was performed as a sequel to our previous cross-sectional study, conducted in 2003. The comparison shows that the prevalence of combined OW/OB has increased nearly twofold (from 18% in 2003 to 33% in 2015) and the prevalence of OB alone has increased nearly threefold (from 3.4% to 9.8%) during the course of 12 years (2003-2015) in Antalya, Turkey. Although there are several reports which have shown a plateau or a decreasing trend of childhood OW and OB in recent years from some countries including the United Kingdom (10), Ireland (3,11), France (12), Sweden (13), Italy (14), Germany (15), Australia (16) and the United States (17) the general prevalence trend for OW and OB is increasing among children and adolescents in both developed and developing countries, as is the case in Turkey (2,4,18). Alper et al (4) showed in a meta-analysis that the overall prevalence of OB in Turkey is 7.3% among school aged children (6.8 % in girls, 7.4 % in boys). However, the prevalence of OW and OB in our study appears to be much higher than in other regions of Turkey (4,5,19,20). A possible cause of this difference may be the fact that the current study is one of the latest in the literature and reflects the upward trend in Turkish OB. A further possible reason may be that the study was conducted in a region with high socioeconomic status. The ratios we report are very high compared to the literature and even higher than those of developed countries (2). 

In our 2003 study, we reported that there was no difference in OB rates between girls and boys, while OW prevalence was higher in girls. This situation has changed. OB has become significantly more common in boys (11.3%) than girls (8.1%) (p<0.05) and that OW prevalence is similar in both sexes (23.9% in boys, 22.5% in girls). Alper et al (4) also reported that the prevalence of OB increased markedly from 1.2% to 6.8% in girls and from 0.3% to 7.4% in boys over a longer period in Turkey; between 1990-1995 and 2011-2015. The trend of increase in boys was also higher than girls in this study which suggests that, in recent years, boys have become more likely to be OB than girls on a national scale. It is not known why the prevalence of OB in boys increases faster than girls. A study from the Netherlands also showed a noticeable increase in the OW and OB prevalence among Turkish children living in the Netherlands although children of Moroccan, Surinamese, South Asian and Dutch descent showed no similar trends. This finding was more pronounced among Turkish boys than Turkish girls with only a mild increase in OB prevalence in the girls from 1999 through 2007 (21). 

When analyzed according to age groups, we observed that the prevalence of combined OW/OB increased rapidly the mid-childhood years and appeared to plateau in late childhood. While the prevalence of OB gradually decreased after age 11, the prevalence of OW did not. Koca et al (22) reported that the prevalence of OB in children under 11 years of age was higher than that of older children in Isparta, a city located in the south-west of Turkey in line with our results. We found that girls reach the highest prevalence of combined OW/OB at 10 years of age while boys arrive at peak prevalence a year earlier. The distributions of combined OW/OB prevalence and OB prevalence alone by age are compatible with global data in girls (2). In the meta-analysis of American data by Wang and Beydoun (18) the highest prevalence of OB in childhood is between the ages of 6-11 years for girls and boys. In another study conducted in the Netherlands among subjects aged 0-21 years, the prevalence of OB and OW was shown to peak between 4-7 years of age (23).

All these studies indicate that the prevalence of OB in children shows its peak during the primary school years. As the highest prevalence of OB appears to occur in the primary school years, targetted preventative and education programmes should be considered for children and parents during these ages and shoud probably be implemented at even earlier ages. In March 2016 the Turkish Ministry of National Education and the Ministry of Health published a joint statement including the list of foods suitable or unsuitable for sale in school canteens. This was a small but positive step towards increasing awareness among children and their parents. However, surveillance and monitoring of trends in the prevalence of OW and OB are required to determine whether such actions are beneficial and to plan future actions.

The LMS method, which depends on the BMI calculation, is generally used to define OB and OW in childhood (8). The age- and sex-specific BMI reference percentile charts derived from our data had higher cutoffs than those of the study conducted by Turkkahraman et al (7) in 2003 and other studies conducted in different regions of Turkey (İstanbul in 2002 and Kayseri in 2008) (19,20). Since the etiology of childhood OB is multifactorial, it is difficult to explain the underlying cause of these differences in BMI cutoff values.

### Study Limitations and Strengths

There are some limitations to this study. Firstly, as this was a sequel to a previous study and since the studies were conducted 12 years apart, it is not possible to show the fluctuation in the prevalence of OW and OB during these years. Secondly, this was a cross-sectional study performed in a relatively limited area of a single city and thus cannot reflect the characteristics of the whole Turkish population. There are also some important strengths to our study. These include the fact that both studies were performed by the same pediatric endocrinology team. Furthermore, all measurements in the study were performed by experienced health personnel, which increases the reliability of results despite inter-observer variation. Another important strength of the study was the reliable comparison of OW and OB prevalence and their trends via two studies with very similar characteristics and two sets of data which were adjusted for age and sex.

## Conclusion

The results of our study demonstrate a striking increase in the prevalence of OW and OB in the city center of Antalya, Turkey in line with data from other pediatric populations. If this trend is replicated nationally then there is a pressing need for both regional and national OB prevention strategies. The effectiveness of these interventions should be measured by on-going surveillance studies and the OB prevalence trend among children should be closely monitored.

## Figures and Tables

**Table 1:**
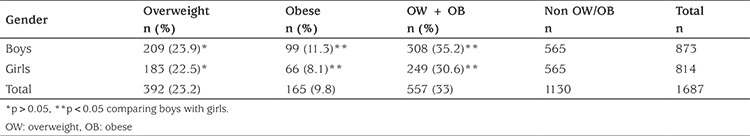
Frequency of overweight and obese children of ages 6-14 years in Antalya in 2015

**Table 2:**
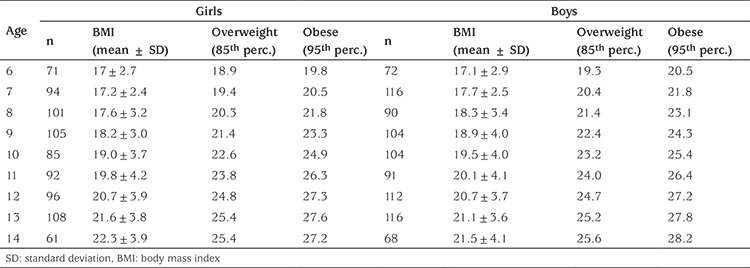
Body mass index percentiles of children aged 6-14 years, in Antalya, Turkey

**Table 3:**
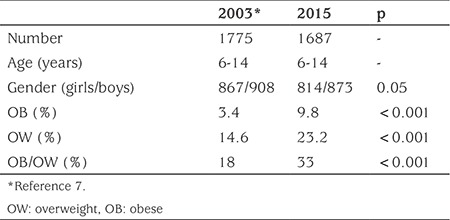
Obesity and overweight figures for 6-14 years old school children in Antalya in 2003 and 2015
